# Assessing a facilitated social network intervention for health outcomes in lonely and socially isolated people: the pragmatic, cluster-randomized PALS trial

**DOI:** 10.3389/fpubh.2026.1701579

**Published:** 2026-03-30

**Authors:** Rebecca Band, Jaimie Ellis, Karina Kinsella, Elizabeth James, Sandy Ciccognani, Katie Breheny, Megan Lawrence, Rebecca Kandiyali, Sean Ewings, Anne Rogers

**Affiliations:** 1 Faculty of Medicine, Health and Life Science, School of Health and Social Care, Swansea University, Swansea, United Kingdom; 2 Faculty of Environmental and Life Sciences, School of Health Sciences, University of Southampton, Southampton, United Kingdom; 3 School of Applied Social and Policy Science, Ulster University, Londonderry, United Kingdom; 4 School of Health, Leeds Beckett University, Leeds, United Kingdom; 5 Population Health Sciences, Bristol Medical School, University of Bristol, Bristol, United Kingdom; 6 Southampton Clinical Trials Unit, University of Southampton, Southampton, United Kingdom; 7 Centre for Health Economics at Warwick, Warwick Medical School, University of Warwick, Warwick, United Kingdom

**Keywords:** community research, loneliness, mental health, pragmatic randomized trial, social isolation

## Abstract

**Introduction:**

Loneliness has received attention in recent years as an important public health issue due to its associations with poorer mental and physical health. This study aimed to assess the effectiveness and cost-effectiveness of a facilitated social network intervention to alleviate loneliness and social isolation in community settings.

**Methods:**

A pragmatic, cluster-randomized controlled trial (RCT) was conducted to compare participants receiving the intervention with a wait-list control group. This trial also included an embedded economic evaluation and took place in two cities in England, UK. The intervention was a facilitated social network tool. It connects people to local community resources to potentially increasing their social involvement. First, participants mapped out and reflected on their personal social networks. Second, they completed a questionnaire to ascertain their preferred activities, interests, and support needs linked to a local resource database. The intervention was delivered by a trained facilitator, either in person or remotely. Community-based organizations (*n* = 44) identified adults at risk of social isolation and/or loneliness. The control group received” usual care” from the organization that recruited them. The primary outcome was the 6-month 12-Item Short Form (SF-12) Health Survey mental health composite score. Physical health, wellbeing, loneliness, social isolation, and collective efficacy were explored as secondary outcomes. Intervention costs, healthcare resource use, quality-adjusted life years (QALYs), and net monetary benefit (NMB) were included in the economic analysis.

**Results:**

A total of 469 adults were recruited between November 2018 and November 2021, with an 8-month in recruitment due to the coronavirus disease 2019 (COVID-19) in 2020 (242 participants in the intervention group and 227 participants in the control group). The results showed no clinically meaningful treatment effect of the intervention on the primary (0.21; 95% confidence interval [CI]: −1.74–2.16; *p* = 0.834) or secondary outcomes when compared to the usual care control group. The economic evaluation indicated no significant difference in QALYs and did not demonstrate cost-effectiveness despite being inexpensive to deliver.

**Discussion:**

The findings indicate that the intervention was not effective under trial conditions. Future interventions aimed at reducing loneliness and social isolation would likely benefit from a multi-step approach that includes tailored psychological, relational, and social components and considers the structural availability of community assets.

**Clinical trial registration:**

https://doi.org/10.1186/ISRCTN19193075, ISRCTN19193075.

## 1 Introduction

Loneliness is a subjective psychological state where there is a discrepancy between desired and perceived levels of support or connectedness, while social isolation refers to a lack of social connections, contact, or participation (1, 2). Although they are distinct psychosocial constructs (3), the impact of loneliness and isolation on wellbeing and the associated health risks represent a significant public health concern (4–6). Both loneliness and social isolation are associated with poor physical and mental health outcomes (7, 8), including general health, wellbeing, and cognition (9, 10), health-relevant behaviors (11, 12) and reduced quality of life (13, 14). Early evidence indicated that the health impact of loneliness may result in a mortality risk greater than that associated with major risk factors such as obesity, alcohol consumption, and cigarette smoking (15–17). Recent studies continue to support the link between loneliness and poorer health outcomes, such as cardiovascular disease (18) and infectious diseases (19). Additionally, loneliness is associated with significant societal costs due to the increased use of health services, such as more general practitioner (GP) appointments, higher rates of emergency hospital admissions, and premature reliance on social care (20–24).

Both loneliness and isolation have gained attention since the coronavirus disease 2019 (COVID-19) pandemic (25, 26), and recent estimates suggest that nearly half of adults in the UK feel lonely occasionally (27). Loneliness is linked to several demographic factors and socioeconomic deprivation in children, young people, and adult populations (5, 28, 29). Although there is accumulating evidence to suggest that loneliness is a significant issue experienced by large portions of the general population (30), at-risk adult groups include older adults, minority communities, and those with long-term mental or physical health conditions (12, 31–34). Factors that lead to reduced social interaction and participation, such as loss of employment, retirement, ill health, and widowhood, are identified as potential contributors to feelings of loneliness and isolation (5, 12, 33, 35, 36).

One effective mechanism to reduce the impact of loneliness is by improving the quality of interpersonal relationships and increasing social participation (5, 37, 38). People report lower levels of distress when they spend more time with people they can rely on for support. A focus on social networks offers the opportunity to move beyond individualized interventions, acknowledging both micro and macro factors that influence health outcomes and behaviors (39), which may be particularly relevant when considering social determinants of health such as loneliness (40). Social network characteristics, such as size and connectivity, and socioeconomic properties and network locality have been found to be associated with loneliness across the population (41–44). A smaller social network size has been associated with a higher risk of mortality related to loneliness (45). Importantly, social networks play a key role in mediating access to resources and facilitating connections to formal and informal support (46).

Social network interventions can improve health outcomes, enhance quality of life, and increase engagement in new activities (47, 48). A diverse and supportive network has been shown to reduce health service costs (49). In line with this evidence, there is a strong rationale for connecting people within their communities to mediate against loneliness. Therefore, this study aimed to evaluate a social network intervention in a community setting. This report presents the effectiveness and cost-effectiveness of the intervention in addressing social isolation and loneliness compared to a wait-list control group.

## 2 Materials and methods

The methods of the Project about Loneliness and Social Networks (PALS) study have been described in detail elsewhere. Briefly, the study was a pragmatic, community-based, cluster-randomized controlled trial (RCT) with embedded health economic evaluation. The study compared the intervention with a wait-list control group for addressing loneliness and social isolation (50).

### 2.1 Population

Eligible participants were aged 18 or older and identified by community-based partner organizations as potentially at risk of loneliness and/or social isolation.

Exclusions included participants who were not living in a community setting, having a terminal illness or other medical condition that affected participation, including a lack of capacity to take part, and anyone who had used the intervention previously.

### 2.2 Randomization and blinding

It was originally planned that each organization would identify two members of the workforce for the study, with one randomly assigned as the trained intervention facilitator and the other as the control facilitator (i.e., facilitator-level randomization, stratified by organization). However, it became clear that randomization needed to be more flexible than originally planned to work across different organizational contexts. The implementation process highlighted that cluster-randomization was not required and that individual participant randomization was achievable. In cases where a facilitator had ongoing contact with potential participants, the facilitator would be randomized (but not participants), and training would be provided only to those assigned to the intervention arm. In cases where there was no potential ongoing contact and, therefore, no risk of contamination, all facilitators were trained, and randomization occurred at the participant level. A member of the research team randomized the participant after baseline data collection. Participants and community partners were not blinded to the allocation of participants.

### 2.3 Procedure

All participants were referred to the study via one of 44 community-based partner organizations located in the South and North-West regions of England. Organizations were approached to collaborate if they were able to identify potential participants, and organizational involvement in the study was flexible for as long as was feasible or until they had exhausted all potential participants [see Ellis et al. (51) for further information regarding the partner organizations and the process of developing specific implementation plans to accommodate specific organizational needs]. All participants were provided with written study documents and were able to ask questions to the research team before providing informed, written consent. Data collection took place at baseline (0 months), 3 months, and 6 months follow-ups; participants were able to complete this on paper or electronically (majority of them completed on paper) and with in-person or telephone assistance from the research team, as required. Intervention group facilitators contacted participants to arrange the intervention delivery, which was in person prior to the coronavirus disease 2019 (COVID-19) outbreak and delivered remotely subsequently. Follow-up assessments were sent by post at 3- and 6-months after enrollment.

Participants were able to complete these independently or with assistance from the research team (with telephone assistance) if required. Follow-up assessments were eligible to be completed in a window of 2 weeks before and 6 weeks following the follow-up date.

### 2.4 Intervention

The PALS intervention for loneliness consisted of an adapted version of the GENIE intervention (48). GENIE was developed in the context of self-management support for long-term conditions, underpinned by evidence for the beneficial properties and mechanisms of social network support for health outcomes (48, 52, 53). However, GENIE was designed to be a generic tool for use beyond supporting long-term physical and mental health conditions. A trained facilitator guides the recipient through the web-based tool consisting of two stages:

#### 2.4.1 Network mapping

The participants were guided to build their network map, which includes the people, places, or things important to them in their daily lives. This uses the concentric-circle technique, where the most important network members are placed nearest to the center, and each subsequent concentric circle represents a lower degree of importance to the participant. Each final map was checked with the participant to ensure its accuracy.

#### 2.4.2 Linking participants to valued activities and support

The second part explores activities or resources in categories prioritized by the participant, linked to their postcode (41). This part of the intervention identifies local resources, sources of support, and relevant community-based groups and activities presented on a Google-generated map.

Participants were given a copy of their social network map and the details of the preferred local activities. Where delivery was remote, this was posted to participants. The intervention group received the intervention at baseline and were offered the opportunity to repeat at 3-month follow-up (updating network maps, reflecting on changes, and exploring additional community groups or activities, if relevant).

### 2.5 Control group

The control group received “usual care” provision from the partner they were linked to, and as such,” usual care” received within the study depended on the organization the participant was recruited through. All control group participants were able to access the intervention after the completion of their 6-month follow-up.

### 2.6 Outcomes

The primary outcome of the trial was mental wellness at 6-month follow-up as measured by the 12-Item Short Form (SF-12) Health Survey mental health composite scale score (MCS) (54). At the time of study development, no previous research had examined social isolation and loneliness outcomes within an RCT. The MCS was selected as the primary outcome because it has been used in studies of community-based interventions with similar populations. Secondary outcomes included physical health (measured by the SF-12 physical health composite scale score; PCS) (54), loneliness [measured by the De Jong Loneliness Scale (55) and the Campaign against loneliness measure (56)], social isolation [measured by the Duke Social Support index (57)], wellbeing [measured by the Warwick Edinburgh Mental Wellbeing scale; SWEMWBS (58)], collective efficacy [measured by the Collective Efficacy Network Scale; CENS (59)] and social support measured via SPA (60).

### 2.7 Unplanned adaptations to PALS delivery during COVID-19

The study’s recruitment pause lasted approximately 8 months due to COVID-19, from March 2020 until November 2020, when recruitment resumed. Due to the ongoing and ever-changing restrictions in place during that time, all facilitator training (for community-based partners) was adapted and delivered through an online platform (Microsoft Teams or Zoom). In addition, facilitators subsequently delivered the intervention remotely (prior to the pause, all intervention delivery had been in-person).

### 2.8 Sample size

The sample size calculation was based on the primary analysis of the comparison of intervention and usual care on the SF-12 MCS at 6 months (54). Previous studies (albeit in different populations) suggested differences of 3–4.7 points on the SF-12 would be clinically meaningful (61, 62). The sample size for this study was based on a difference of 4 points, a standard deviation of 10.4 [based on a previous study in socially isolated older people (63)], 80% power, and a type I error rate of 5%, resulting in 216 people (108 per arm). As the study was set up to be cluster-randomized, the sample size calculation accounted for possible intra-cluster correlation (ICC); the calculation also accounted for the model with two facilitators per organization and 12 participants per facilitator. No estimates for the ICC were found in the literature that were directly relevant to the current study population; however, previous studies have generally shown low ICCs for mental health scores from SF-12 and SF-36 (0.032 and below) across GP practices (39, 64); a value of 0.05 was set for the ICC to prioritize avoiding an under-powered study with the view that this was likely to be more conservative. The adjusted sample size was *n* = 335, which, once accounting for a potential drop-out of 15% (40), resulted in a total sample size of 394 participants (197 per group). Following the recruitment pause, further adjustments were made to the sample size to account for unplanned changes due to COVID-19 (i.e., the high loss to follow-up in 2020). The original complete case sample size remained at 335, with the total sample size adjusted to *n* = 453, assuming a 15% drop-out rate during the post-pandemic restart.

### 2.9 Patient and public involvement

Community-based partner organizations were involved in the development of the study and throughout. During the study, partner organizations were involved in agreeing on participant identification, recruitment methods, intervention delivery, and resolving implementation issues. Patient and public involvement (PPI) representatives were involved in quarterly study management group meetings.

### 2.10 Statistical analysis

The statistical analysis plan was developed before the final analysis (see Band et al. 50 for further details). Intercurrent events (defined per the International Council for Harmonization (ICH) E9 (R1) Statistical Principles for Clinical Trials [ICH E9 (R1)] Addendum) are listed in Table 1 alongside the strategy and reason (65). The pragmatic design of the study, selected to inform intervention delivery and scalability in real-world settings, informed the statistical analysis strategies.

**Table 1:** Intercurrent events and strategies for the primary estimand.

Intercurrent event	Strategy	Rationale/justification
Intervention not delivered according to protocol (including: not initiated; withdrawal from using PALS intervention)	Treatment policy	Likely to reflect the reality of delivering and engagement with the PALS intervention in practice
Use of any other approaches to supporting mental health (competing or complementary interventions)	Treatment policy	Other approaches will generally be available outside the trial, and the aim is to assess PALS in this context
Death	Principal stratum	Deaths were expected to be very low and will therefore be excluded from the analysis due to the assumed minimal impact on conclusions
COVID-19 pandemic (impacting intervention delivery, and participant mental health)	Treatment policy	See discussion below

Reproduced from “Intercurrent events and strategies for the primary estimand” by Rebecca Band, Karina Kinsella, Jaimie Ellis, Elizabeth James, Sandy Ciccognani, Katie Breheny, Rebecca Kandiyali, Sean Ewings and Anne Rogers, licensed under CC BY 4.0.

Analyses were carried out in Stata version 16.0.

It was decided that while the COVID-19 pandemic may affect mental health, the proposed treatment mechanism should be largely unaffected by intervention delivery or the opportunities for social participation available to participants, thereby not affecting the treatment effect. The pandemic was therefore considered under the treatment policy strategy, with a single treatment effect covering the pre- and post-pandemic periods (50). The timing and impact of the pandemic meant that there were approximately three equally sized participant groups: (1) those recruited and followed up before COVID-19; (2) those recruited before COVID but followed up during the first lockdown; (3) those recruited and followed up after the study restart in November 2020 (and subject to varying restrictions). Therefore, the treatment policy decision was also taken pragmatically based on this, and the primary estimand was the treatment difference (PALS intervention vs. no PALS intervention) for all randomized participants who would not die, regardless of how the intervention was delivered and the participants’ engagement with it, and regardless of the COVID-19 pandemic (50). All secondary outcomes were considered to have the same intercurrent events and would be dealt with using the same strategies.

Recruitment was extended during the study to ensure that a complete case analysis was adequately powered. It was believed *a priori* that only outcome data would be missing (models were planned to include terms for organization, facilitator, baseline score of the outcome measure, and time point, which are highly unlikely to be missing). Hence, following the guidance of Jakobsen et al. (66), a complete case analysis was planned, even though this strategy did not necessarily align with the primary estimand; however, reasons for withdrawal were not collected systematically or consistently, making it difficult to fully explore reasons for missingness and the potential impact on study conclusions.

Baseline data are presented based on whether primary outcome data were collected. Any differences between those who did and did not provide primary outcome data were assessed informally (50).

#### 2.10.1 Primary endpoint

The difference in the SF-12 (version 2) MCS score between the intervention and control groups at 6-month follow-up was estimated using a linear mixed-effects model. The primary model was anticipated to include outcomes at 3 and 6 months, with participant and organization included as random effects. It was planned that, if the mixed model did not converge, a model with only 6 months as the outcome would serve as the primary analysis. For the complete case analysis, it was planned that if missing data rates were higher for 3 months than that for 6 months, a model with only 6 months as the outcome will be considered the primary analysis (this situation was thought possible due to the nature of the impact of the first UK COVID-19 pandemic lockdown on the study; in this instance, it was planned that participant and time will no longer be included in the model). Time (as a categorical variable) and baseline SF-12 MCS score were included as fixed effects. An unstructured covariance matrix for the random effects was planned, and the model was to be fit using restricted maximum likelihood (REML). It was planned to include the facilitator as a random effect; however, delivery was largely carried out by the trial team, so it was felt that the facilitator would not be an important factor.

Scoring for the SF-12 followed the User’s Manual for the SF-12v2 Health Survey, Third Edition. Missing data was handled using the built-in Full Missing Score Estimation option; briefly, missing items on a single questionnaire may be imputed in a domain with more than one question. This approach was also used for the secondary endpoint of the SF-12 Physical Composite Score (PCS).

### 2.11 Economic analysis

The economic analysis aimed to assess whether PALS was a cost-effective intervention for individuals at risk of loneliness, compared with usual care, delivered in a community setting. A within-trial cost-utility analysis was conducted over a 6-month time horizon. As the time horizon was less than 12 months, no discounting was conducted. The economic evaluation sought to address the estimated resource use and cost of delivering PALS, the effect of PALS on health-related quality of life (SF-6D) (67) and capability-wellbeing (ICEpop CAPability measure for Adults [ICECAP-A]) (68) and the cost-utility, in terms of net monetary benefit (NMB), of PALS.

#### 2.11.1 Costing the intervention

Resource use for both facilitator training in the intervention and subsequent delivery was estimated based on within-trial logs completed after each activity, and on the recorded duration, nature, and number of staff attending the sessions, as well as whether it was face to face (and, if so, the facilitator’s travel was recorded) or virtual.

Participants’ healthcare resource use was collected using a *de novo* resource use questionnaire that included health and social care resources (e.g., medical or mental health visits or contacts) and informal care (e.g., from family and friends) at baseline, 3, and 6 months. Questions regarding personal expenditure on activities (which might be influenced by the intervention) were included, and a total activity cost was calculated for each participant.

Resource use was valued using appropriate UK unit costs or participant valuations, estimated at the time of the study delivery (2019–2021). Table 2 outlines the unit costs and assumptions. National Health Service (NHS) National Cost Collection data (69) was used to value hospital resource use (e.g., Accident and Emergency [A&E] visits and outpatient attendances). Primary care and social care costs (e.g., GP visits) were valued using the Personal Social Services Research Unit (PSSRU) Unit Costs of Health and Social Care (70). The total number of prescriptions was also collected for each participant, and unit costs were per prescription (see Supplementary File 1).

**Table 2:** Means and SDs of SF-12 mental composite score (MCS) by group and time point, and group differences over time.

Statistic	Usual care	Intervention
Baseline – *N*	219	241
Mean (SD)	44.6 (11.5)	43.6 (12.4)
3 months – *N*	135	155
Mean (SD)	44.7 (11.5)	43.6 (12.0)
Mean difference (95% CI)*	–	−1.27 (−3.53–0.99)
*p*-value	–	0.271
6 months – *N*	169	180
Mean (SD)	41.9 (11.6)	42.7 (12.0)
Mean difference (95% CI)	–	0.21 (−1.74–2.16)
*p*-value	–	0.834

SD, standard deviation; CI, confidence interval. *Regression based on *n* = 289 due to missing organization variable for usual care group participant. Reproduced from “Means and SDs of SF-12 MCS by group and time point, and group differences over time” by Rebecca Band, Karina Kinsella, Jaimie Ellis, Elizabeth James, Sandy Ciccognani, Katie Breheny, Rebecca Kandiyali, Sean Ewings and Anne Rogers, licensed under CC BY 4.0.

Participants reported the number of hours of unpaid care received from friends and family using a single question. The value of this time was estimated using the opportunity cost approach (71), using the National Living Wage 2019–2020 (72).

##### 2.11.1.1 Outcomes

The primary analysis for the economic evaluation uses the quality-adjusted life years (QALYs), derived from utility scores, obtained using the SF-6D algorithm (73), which uses SF-12 data collected at baseline, 3, and 6 months. A secondary economic outcome was the ICECAP-A measure of capability wellbeing (68). UK tariff values (74) were used to generate utilities and years of sufficient (YSC) and years of full capability (YFC).

QALYs were generated using the area under the curve approach (75). Years of sufficient capability were generated using the method proposed by Mitchell et al. (76) and the threshold of sufficient capability defined by Kinghorn (77) (response profile of 33,333). Table 3 reports the costs included in each perspective.

**Table 3:** Costs included in each perspective.

Costs	Perspective	
	Public sector	Societal
Intervention delivery	Included	Included
Health care resource use	Included	Included
Formal care	Included	Included
Informal care	Excluded	Included
Participant activities	Excluded	Included

Reproduced from “Costs included in each perspective” by Rebecca Band, Karina Kinsella, Jaimie Ellis, Elizabeth James, Sandy Ciccognani, Katie Breheny, Rebecca Kandiyali, Sean Ewings and Anne Rogers, licensed under CC BY 4.0.

##### 2.11.1.2 Analytic approach

A net benefit approach was applied to the within-trial economic evaluation, where the cost/QALY data were transformed into a continuous variable. This allows for correlation between both endpoints and easier manipulation of the data, including within a multilevel framework. Incremental costs, outcomes, and net NMB were analyzed using multilevel linear models (adjusted for clustering by organization). NMB plots and cost-effectiveness acceptability curves (CEACs) were used to explore uncertainty around NMB. In addition to our *a priori* complete case primary analysis, imputation was carried out to address the substantial amount of missing data. We used the multiple imputation framework, as it is considered preferable to simpler forms of imputation, which can underestimate uncertainty and fail to account for all the observed data (78). Missing data were assumed to be missing at random and were imputed at the component level. Costs and SF-12 scores were imputed jointly using chained equations and predictive mean matching; our imputation model matched our analysis model, with age added as an auxiliary variable. Analyses were conducted in Stata 16.1. It was decided that sub-group analysis would not be conducted. We adopted an explanatory focus in interpreting the economic results in light of what we learned during intervention delivery (79, 80).

### 2.12 Trial registration and approvals

The PALS study is registered as ISRCTN19193075. Ethical approval for the study was received from the South Central – Berkshire Research Ethics Committee (reference number: 18/SC/0245) and from the Health Research Authority (HRA) in June 2018. Four substantial amendments were made during the study; these were to amend the exclusion criteria, to adjust the randomization process set out in the original protocol, and to pause and restart the study due to the COVID-19 pandemic.

## 3 Results

### 3.1 Participant characteristics and intervention delivery

**Figure 1:**
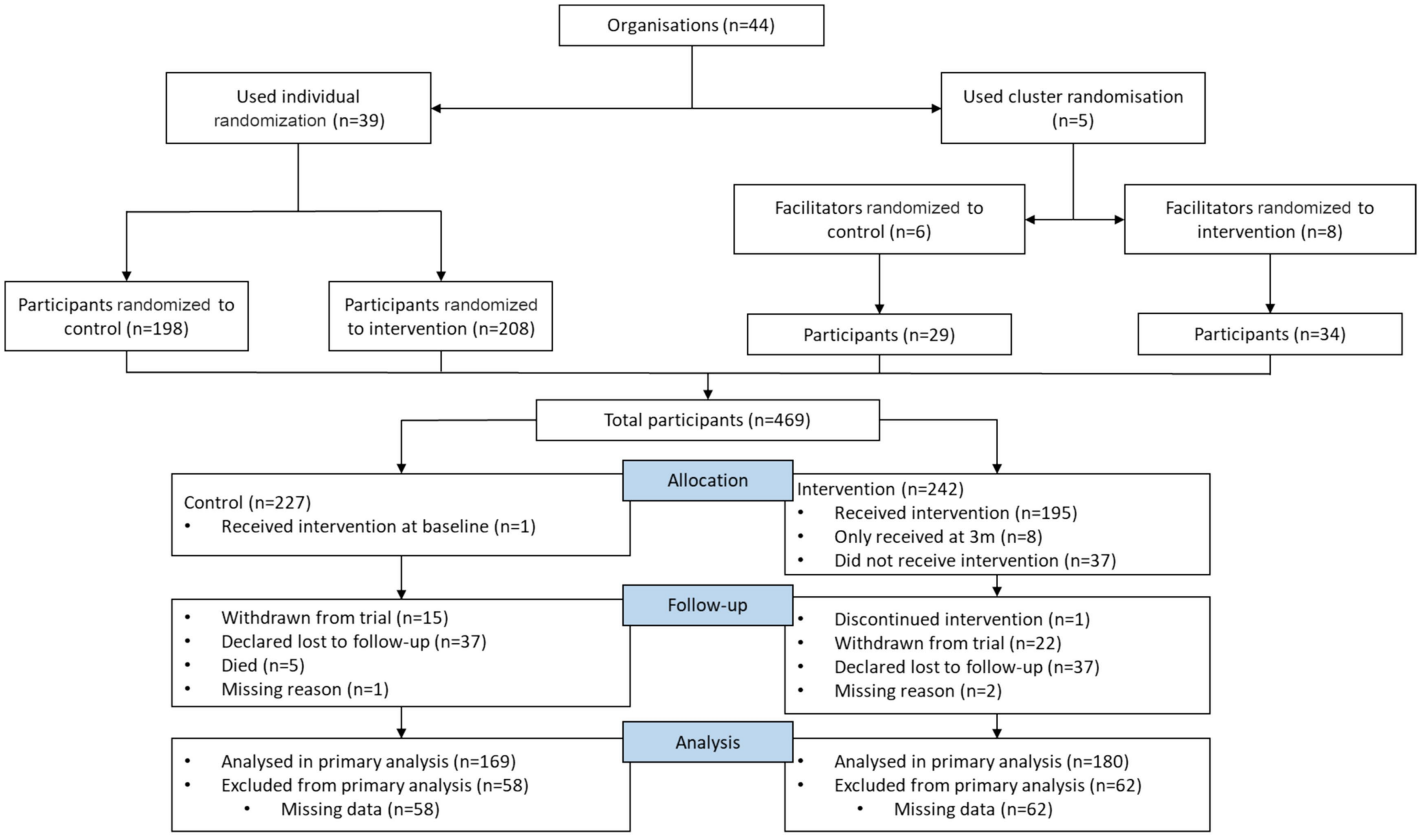
Flowchart of PALS trial.

Participants were recruited into the study over a 32-month period between November 2018 and November 2021 (which included an 8-month pause in 2020 due to COVID-19). In total, 656 potential participants were referred to the study team, and 469 (72%) completed baseline assessments. The mean recruitment rate was 15 participants per month; however, this was almost halved following the onset of the COVID-19 pandemic (average 19 per month pre-pandemic vs. 10 per month post-pandemic). In total, the study team delivered the intervention to 138 participants (71%). One hundred and twenty participants withdrew from the study (26%), with similar numbers across both trial arms (*n* = 62 intervention participants vs. *n* = 58 control participants). Approximately 68% of all study withdrawals (*n* = 82) occurred between March and August 2020 (during the height of COVID-19 pandemic restrictions), with 74 (62%) lost to follow-up. The CONSORT guidelines were followed for reporting cluster-randomized trials. Figure 1 outlines participant flow through the study.

Participants ranged 19–95 years old, and the median age of participants was 65 years (see Table 4 for baseline characteristics of participants). The majority of the participants were female (70%), of white ethnicity (92%), and just over half lived alone (52%). Approximately 80% of participants were not working at the time of study entry. Over 75% of participants reported a household income of less than £26,000 per annum (77%), with 55% of the sample reporting a household income of less than £15,600 per annum [compared with a UK national household average income of £31,400 in 2021 (81)]. The sample mean index of multiple deprivation (IMD) decile score was 4.18 (standard deviation [SD] = 2.76).

**Table 4:** Baseline characteristics of participants in the control and intervention groups in the PALS Study.

Characteristic		Control (*n* = 227)	Intervention (*n* = 242)
Age (years) – median (LQ, UQ)^a^		65.0 (48.0, 76.0)	64.0 (47.5, 75.5)
	Missing	8 (4%)	2 (1%)
Sex – *N* (%)			
	Male	70 (31%)	69 (29%)
	Female	157 (69%)	173 (72%)
Ethnicity – *N* (%)			
	White	209 (92%)	224 (93%)
	Other	18 (8%)	18 (7%)
Highest education level – *N* (%)			
	Primary school	5 (2%)	3 (1%)
	Secondary school	88 (39%)	105 (43%)
	College	79 (35%)	61 (25%)
	Higher education	51 (22%)	71 (29%)
	Missing	4 (2%)	2 (1%)
Working status – *N* (%)			
	Full-time	18 (8%)	18 (7%)
	Part-time	21 (10%)	21 (9%)
	Retired	108 (48%)	117 (48%)
	Unemployed	20 (9%)	25 (10%)
	Unable to work	52 (23%)	53 (22%)
	Education or training	3 (1%)	5 (2.%)
	Carer	0	1 (0.4%)
	Missing	5 (2%)	2 (1%)
Living status – *N* (%)			
	Owner-occupied	111 (49%)	119 (49%)
	Rented from the council or a housing association	76 (34%)	90 (37%)
	Rented from a private landlord	30 (13%)	26 (11%)
	Temporary accommodation	1 (0.4%)	2 (1%)
	Other	5 (2%)	3 (1%)
	Missing	4 (2%)	2 (1%)
Living alone – *N* (%)			
	Yes	115 (51%)	127 (3%)
	No	108 (48%)	113 (47%)
	Missing	4 (2%)	2 (1%)
De Jong scale – mean (SD)		3.1 (2.0)	3.4 (2.1)

a LQ, lower quartile; UQ, upper quartile; SD, standard deviation. Adapted from “Baseline characteristics of participants in the control and intervention groups” by Rebecca Band, Karina Kinsella, Jaimie Ellis, Elizabeth James, Sandy Ciccognani, Katie Breheny, Rebecca Kandiyali, Sean Ewings and Anne Rogers, licensed under CC BY 4.0.

The study groups were well matched (*n* = 227 in the control arm and *n* = 242 in the intervention arm), with no major differences between the arms. Five usual care group participants died during the trial. and are not included in the following primary or secondary analyses, leaving a total *n* = 464 (as pre-specified in the statistical analysis plan). Nineteen participants in the intervention group (8%) received no intervention at either time point (i.e., at baseline or 3 m follow-up) due to the participant being uncontactable (*n* = 15, 79%), because they did not want the intervention (*n* = 1; 5%), or because of health issues (*n* = 3, 16%). Five participants did not receive the intervention at baseline but did receive it at 3-month follow-up due to COVID-19 restrictions (*n* = 4, 80%) or health issues (*n* = 1, 20%). One participant in the control group received the intervention in error (but was included in the analysis).

### 3.2 Main trial results

After 6 months, approximately one quarter of questionnaires were missing at the primary time point (control group: 24%; intervention group: 26%). In view of the higher rates of missingness at 3 months (control group: 39%; intervention group: 36%), the primary analysis reverted to fitting a mixed effects model for SF-12 MCS at 6 months (as pre-specified). The primary analysis of the group comparison at 6 months is presented in Table 5. Results at both time points indicated no meaningful treatment effect (with a Minimal Clinically Important Difference [MCID] of 4 points on the SF-12 MCS scale). Similarly, no or little treatment effect (whether harmful or beneficial) was observed by exploring the estimated mean differences between groups and corresponding confidence intervals (CIs). Baseline SF-12 MCS was also fitted using restricted cubic splines (five knots, in the default positions using Stata command *mkspline*) to allow for a nonlinear relationship with the outcome; this led to minimal change in the results (treatment effect: 0.21, with 95% CI: −1.74–2.17; *p* = 0.832). When using a linear mixed effects model of 3- and 6-month SF-12 MCS data combined (using time as a fixed categorical effect and participant as a random effect nested in organization), the treatment effect was estimated to be −0.16 (95% CI: −1.90–1.58; *p* = 0.858), leading to a similar conclusion of no or limited harmful or beneficial effect of treatment.

**Table 5:** Quality-of-life and wellbeing capability tariff scores, QALYS, years of sufficient and full capability, and costs.

Measure (control, *n*; intervention, *n*)	Usual care	Intervention arm	Unadjusted	Adjusted for baseline level (QALYs only) and organization random effects^a^	Multiple imputation was adjusted for baseline and organization random effects
	Mean (SD) score		Mean difference (95% CI)		
QALYs (95,119)	0.315 (0.069)	0.319 (0.071)	0.004 (−0.015 to 0.022)	0.002 (−0.012 to 0.007)	−0.028 (−0.054 to −0.001)
Years of sufficient capability (115,135)	0.389 (0.010)	0.399 (0.009)	0.010 (−0.017 to 0.036)	−0.00007 (−0.013 to 0.0135)	NR
Years of full capability (115,135)	0.348 (0.097)	0.360 (0.109)	0.012 (−0.014 to 0.038)	0.002 (−0.011 to 0.015)	NR
Public sector cost, £ (185,195)	691.84 (1,009.81)	744.26(912.55)	52.42 (−178 to 283.08)	£88.96 (95% £-132.52–£310.45)	382.07 (−72.33 to 836.48)
Net monetary benefit, £ (95,119)	8961.87 (2326.10)	8815.95 (2711.37)	−145.92 (−845.11 to 553.26)	£-443.72 (95% CI £-1060.38–172.93)	−5,515 (−9980.40 to −1051.26)

a Output reports confidence interval (parametric) from a mixed model, which is adjusted for baseline level and with a random effects term for organization. Reproduced from “Quality-of-life and well-being capability tariff scores, QALYs, YSC and YFC and costs” by Rebecca Band, Karina Kinsella, Jaimie Ellis, Elizabeth James, Sandy Ciccognani, Katie Breheny, Rebecca Kandiyali, Sean Ewings and Anne Rogers, licensed under CC BY 4.0.

The treatment effect at 6 months was further estimated in subgroups using interactions; these subgroups were based on Organization and baseline demographics. While the smaller sample sizes mean no firm conclusions can be drawn, there was no strong evidence to suggest a variable effect across demographic categories. There was a wide range of effects by organization (from approximately −20 to +8.5). However, this may be explained by the small sample sizes within some of the organizations.

#### 3.2.1 Secondary endpoints

The data is compatible with no or little meaningful treatment effects for the secondary endpoints at 3 and 6 months (see Supplementary File 2).

### 3.3 Economic evaluation

#### 3.3.1 Intervention costs

The mean intervention cost per participant was £52.65.

#### 3.3.2 Resource use

Participants in the intervention group received more professional care at 6 months, but informal care was similar between arms. For participants who provided information on activities, there were no differences in the mean number of activities at each time point (baseline mean: 2.28 for usual care and 2.34 for intervention participants), with both groups showing small increases at 3 months (Supplementary File 3). At 6 months, there was no evidence of increased activity in either group; however, usual care reported activities below baseline levels. Resource use, with missing data, was high across both groups and all time points.

In total, 265 (57%) of participants completed the NHS and Personal Social Services (PSS) resource use questions at both time points (see Supplementary File 3). Mean costs were higher in the intervention arm compared to the control (£744.26 intervention; £691.84 control), and from a public sector perspective, the total was £190,834. When societal costs are included (informal care and out-of-pocket activities), the intervention costs remained higher (intervention mean cost of £652.89, control mean of £566.48), but this is due to substantial missing data (27% overall participant -completeness).

#### 3.3.3 Preference-based outcomes

There was a negligible, non-significant difference in QALYs (95% CI: −0.012–0.007), years of sufficient capability (YSC), and years of full capability (YFC) after adjustment for clustering by organization. The incremental cost was £88.96, which favors usual care but is a non-significant result (95% £-132.52–310.45).

#### 3.3.4 Primary economic analysis—complete case

Two hundred and fourteen participants (46%) contributed both QALY and cost data for the primary net monetary benefit analysis of public sector costs. A comparative analysis of public sector and societal costs is provided in Table 5. The incremental net-monetary benefit (intervention vs. usual care) was £-443.72 (95% CI: £-1,060.38–172.93) after adjustment for clustering by organization. This negative symbol signifies an incremental net monetary benefit (iNMB) for the intervention that favors usual care, but the confidence interval overlaps zero, showing that this is non-significant. To consider decision uncertainty as the threshold value of the willingness-to-pay (WTP) threshold varies, we additionally present incremental net monetary benefit (iNMB) plots (see Figure 1 in Supplementary File 4). This shows that the incremental net benefit (iNMB) was just below zero at lower threshold willingness-to- pay values and positive thereafter, suggesting that the results for PALS are fairly equivocal compared with usual care, albeit slightly less preferred. The INMB 95% confidence intervals indicate a high degree of uncertainty surrounding the results, which is greater at higher willingness-to-pay thresholds. This is anticipated as a higher WTP threshold will weight changes in QALYs more, magnifying the uncertainty.

A cost-effectiveness acceptability curve was created, showing an upward slope that leveled off at higher WTP thresholds. At thresholds of £20,000 and £30,000 per QALY, the probability that PALS is cost-effective is 35 and 40% cost-effective, respectively (see Figure 2 in Supplementary File 4).

##### 3.3.4.1 Secondary economic analysis

Due to small differences in costs and negligible, non-significant differences in incremental YFC and YSC, the calculation of the cost per extra year of full/satisfactory capability was not conducted. With multiply imputed data, we found the uncertainty in INMB remained wide, allowing both positive and negative values, and increased as the WTP threshold increased (see Figure 3 in Supplementary File 4). In contrast to the complete case analysis, the probability that PALS is cost-effective was 58 and 60% at the £20 k and £30 k thresholds, respectively.

## 4 Discussion

This study aimed to assess the effectiveness and cost-effectiveness of a facilitated social network intervention for alleviating loneliness and social isolation for adults within community settings in the UK. The findings indicate that there was no significant impact on participant outcomes, including mental and physical health, loneliness, and isolation. Additionally, while the intervention was inexpensive to deliver at around £53 per person, there was no evidence of cost-effectiveness.

However, the findings highlighted that adults across the life course experienced loneliness and social isolation. Some 40% of participants in the study reported MCS and PCS scores “well below” population benchmarks, with mean scores notably lower than “healthy” population scores that were comparable to cancer patients (82). The findings also highlight that many participants’ sociodemographic circumstances increased their vulnerability, such as living in deprived areas, not working or receiving low incomes. There is a strong connection between poverty and the risk of social isolation (83) and with a close link between mental health, physical health and risk of experiencing loneliness and social isolation, this study illuminates the vulnerability of individuals who experience loneliness or isolation. Our findings add to the accumulating evidence that loneliness is a significant problem associated with poor socioeconomic, demographic and community-based factors (29, 84, 85).

This study selected the SF-12 mental health subscale as a primary outcome as a proxy for loneliness due to no prior evidence exploring loneliness as an outcome in an RCT at the time of study development (86). Despite accumulating evidence of the impact of loneliness on poor mental health and wellbeing (10, 34), we suggest that the levels of deprivation and poor health seen across our sample may have impacted the potential to capture changes in outcomes, such as mental health or loneliness, especially across a global pandemic, which we know also impacted these outcomes (12, 44, 87–92). This article provides detailed estimates of population-level outcomes for widely cited and relevant measures among adults who are lonely. We believe that research exploring what constitutes a meaningful change in loneliness on existing, validated scales and the likely time scales over which change might be observed would be a useful direction for future research. Additionally, the development of sensitive measures of loneliness that capture meaningful change over time would also be beneficial. Our findings also indicate that interventions to address loneliness must also consider and account for the social determinants of health (40).

The key priority of the intervention was to connect people to personalized and local community-based activities. The acceptability of the intervention to both community organizations and individuals was high, yet there were barriers to delivering an intervention in a community setting (51, 93). One barrier was the community partner organizations’ ability to deliver the intervention as planned through their own organizational structures due to limited capacity (51, 86, 93). However, as around 70% of the intervention facilitation was delivered by highly trained members of the research team, we are confident that the intervention fidelity was good (94). However, a further barrier identified in the study was the lack of community-based activities for people to engage with, difficulties with transport, and the cost of social participation, which was reflected in low participation rates across participants and all time points. These findings suggest that participant willingness to engage with local community-based activities did not translate into increased engagement and uptake with community resources. These findings echo evidence suggesting that community-based initiatives require connections and activities to be very local for those who are marginalized and lack resources to connect with them (95). The availability and affordability of very localized community assets and resources (i.e., transport) are key drivers of success and sustainability for interventions embedded within community settings (94). We therefore suggest that, for people in deprived areas or circumstances, interventions must include greater assistance in connecting with the local resources, increased resources for these areas, or subsidized (or free) transport schemes to access community-based resources in other areas.

Our qualitative and process analyses also provided evidence for offering psychological support prior to attempting to increase community or social engagement. Previous work suggested that a focus on psychological factors and their role in loneliness is important (96, 97), and some participants requested that additional support to overcome the perceived barriers (i.e., getting to activities or having a buddy to accompany them for the first few times) would have been beneficial, especially for individuals who have been lonely and isolated for some time. Ideally, this would be tailored to each user’s unique circumstances and needs.

Therefore, future initiatives focused on community-based interventions would likely benefit from prioritizing the development of broader community capacity to build community assets, alongside individual outcomes. Social prescribing may provide some capacity to establish links on an ongoing basis with a broad range of social interventions in local communities (98).

### 4.1 Strengths and weaknesses

To our knowledge, this is the first community-based trial addressing loneliness conducted within the UK, and we were able to successfully work with a broad range of community-based partners across two regions in England, who referred over 650 potential participants to the study team. Our aim was to ensure methodological rigor while remaining pragmatic and flexible, and to be responsive to the local contexts, which, in turn, would inform the process evaluation of the potential scalability of an intervention such as this (51, 93). Although the RCT is considered to be the gold standard in terms of evidence generation, we propose that interventions addressing loneliness and social isolation may be better evaluated using alternative methods. The ever-changing and unpredictable nature of research in community contexts makes it difficult to impose rigid RCT methodologies. We aimed to work pragmatically and in partnership with communities in this study, which meant we were able to be flexible in the rapidly changing landscape during the pandemic. However, it was not without considerable challenges across the board, and the impact on study findings is difficult to assess. As a result of pandemic disruptions, the study had high levels of missing data at 6 months (20–30% across outcomes), although the methods used to handle missing data did not change the trial’s conclusions. We monitored participant characteristics before and after the pandemic. Despite no obvious differences in those recruited following the pandemic onset, we cannot account for the population-level changes in loneliness and poor mental health observed across this period, nor for the impact that the suspension of in-person community activities and resources may have had on the study.

To our knowledge, this is the first economic evaluation of a social network mapping intervention in the community setting alongside a full trial [although previous economic evaluations of similar trials have been undertaken (47)]. A limitation of our economic evaluation was missing data—net monetary benefit was completed by less than 50% of the sample, and although our analyses exploring missingness did not identify sources of systematic bias, there is the possibility that unmeasured variables introduced bias into the results. Both the complete case and multiply imputed analyses reveal a high degree of imprecision indicated by the wide confidence intervals on net monetary benefits.

### 4.2 Conclusion

The findings presented here do not provide strong evidence of the efficacy of the PALS intervention to address the complexities of mental health, loneliness, and social isolation. Future interventions may benefit a multi-step approach that provides tailored psychological, relational, and social components. Future intervention evaluations should consider fidelity, participant engagement, and the use of sensitive loneliness outcome measures in their study designs. Implications from this study suggest a need for multi-level interventions that include community assets, neighborhood context, and structural barriers such as transportation and accessibility, alongside elements that build social skills, confidence, and resilience. Engagement of participants may be enhanced through community connector roles and the involvement of a broader range of anchor institutions beyond voluntary organizations to support recruitment and participation. Finally, the use of multidimensional measurement tools capable of detecting meaningful change in target populations should be prioritized.

## Data Availability

The datasets presented in this study can be found in online repositories. The names of the repository/repositories and accession number(s) can be found at: https://library.soton.ac.uk/researchdata/storage.
