# Supplementary material for: Assessing a facilitated social network intervention for health outcomes in lonely and socially isolated people: the pragmatic, cluster-randomized PALS trial
Source: Front Public Health. 2026 Mar 30;14:1701579. doi: 10.3389/fpubh.2026.1701579 (PMC13073093; doi:10.3389/fpubh.2026.1701579)
Supplement: Supplementary file 1 [file Supplementary_file_1.docx]

**Supplementary File 1: Description and sources of unit costs.** Reproduced from "[Description and sources of unit costs](https://www.journalslibrary.nihr.ac.uk/phr/WTJH4379)" by Rebecca Band, Karina Kinsella, Jaimie Ellis, Elizabeth James, Sandy Ciccognani, Katie Breheny, Rebecca Kandiyali, Sean Ewings and Anne Rogers, licensed under [CC BY 4.0](https://creativecommons.org/licenses/by/4.0/deed.en).

| Resource | Description | Unit cost | Source |
| --- | --- | --- | --- |
| Trainer’s time | Local Authority Health Trainer Grade 7  £30,394.34. Gross employee costs including 3% pension 13.8% NI  1,599 hours per year | £19.01 per hour | Local authority advertised position |
| Facilitator’s time | Wellbeing Recovery Worker  £26,812.08 Gross employee costs including 3% pension 13.8% NI  1,599 hours per year | £16.77 per hour | Advertised role at a recruiting organization |
| Trainer/ Facilitator Travel | HMRC car milage rate | £0.45 per mile | HMRC(56) |
| GP appointment (face to face) | Per surgery consultation lasting 9.22 minutes. With qualification and including direct care staff costs | £39 | Unit Costs of Health & Social Care 2020 (53) |
| A&E visit | Accident & Emergency, Outpatient attendance | £133 | 2019/2020 National Cost Collection (National schedule of NHS costs) (52) |
| Primary care nurse | Nurse (GP practice Band 5). Per surgery consultation lasting 9.22 minutes. £63 per hour of patient related work | £9.68 | Unit Costs of Health & Social Care 2020 (53) |
| Mental health care professional (psychologist, counsellor, mental health nurse) | Clinical psychologist, Counsellor specialist (Afc Band 7) Hospital-based scientific and professional staff. £63 per hour. Assume 1 contact is 1 hour | £63 | Unit Costs of Health & Social Care 2020 (53) |
| Social worker | Social worker (adult services) Unit cost per hour including qualifications. Assume 1 contact is an hour | £51 | Unit Costs of Health & Social Care 2020 (53) |
| Prescription | Prescription costs per consultation (actual cost) | £30.90 | Unit Costs of Health & Social Care 2020 (53) |
| Hours of formal care | Home care worker. Face-to-face: Based on the price multipliers for independent sector home care provided for social service. £30 per hour | £30.00 | Unit Costs of Health & Social Care 2020 (53) |
| Hours of informal care | National living wage (25 and over) per hour | £8.21 | National Minimum Wage and National Living Wage rates April 2019-March 2020 (55) |
| Out-of-pocket participant activity costs | Incurred cost | variable | Self-report, based on individual participant data |
