# Supplementary material for: Assessing a facilitated social network intervention for health outcomes in lonely and socially isolated people: the pragmatic, cluster-randomized PALS trial
Source: Front Public Health. 2026 Mar 30;14:1701579. doi: 10.3389/fpubh.2026.1701579 (PMC13073093; doi:10.3389/fpubh.2026.1701579)
Supplement: Supplementary file 2 [file Supplementary_file_2.docx]

**Supplementary File 2: Means and SDs of the secondary endpoints by group and timepoint, and group differences over time.** Reproduced from "[Social network intervention for loneliness and social isolation in a community setting: the PALS cluster-RCT](https://www.journalslibrary.nihr.ac.uk/phr/WTJH4379)" by Rebecca Band, Karina Kinsella, Jaimie Ellis, Elizabeth James, Sandy Ciccognani, Katie Breheny, Rebecca Kandiyali, Sean Ewings and Anne Rogers, licensed under [CC BY 4.0](https://creativecommons.org/licenses/by/4.0/deed.en).

| Measure |  | Usual Care | Intervention | Mean difference  (95% CI) |
| --- | --- | --- | --- | --- |
| SF12 PCS | Baseline – N | 219 | 239 | - |
|  | Mean (SD) | 42.6 (13.4) | 43.9 (13.6) | - |
|  | 3 months – N | 133 | 155 | - |
|  | Mean (SD) | 39.5 (12.7) | 42.2 (12.4) | 1.47  (-0.37 to 3.31) |
|  | P-value | - | - | 0.118 |
|  | 6 months – N | 169 | 180 | - |
|  | Mean (SD) | 40.9 (13.5) | 41.5 (13.3) | -0.69  (-2.48 to 1.10) |
|  | P-value | - | - | 0.451 |
| Warwick-Edinburgh Mental Wellbeing Scale | Baseline – N | 217 | 239 |  |
|  | Mean (SD) | 21.1 (4.0) | 21.4 (4.5) |  |
|  | 3 months – N | 129 | 148 |  |
|  | Mean (SD) | 21.4 (4.9) | 21.8 (5.0) | -0.18  (-1.07 to 0.70) |
|  | P-value | - | - | 0.688 |
|  | 6 months – N | 158 | 175 |  |
|  | Mean (SD) | 21.0 (4.9) | 21.4 (4.4) | -0.39  (-1.14 to 0.36) |
|  | P-value | - |  | 0.310 |
| De Jong Loneliness scale | Baseline – N | 211 | 231 |  |
|  | Mean (SD) | 3.1 (2.0) | 3.4 (2.1) |  |
|  | 3 months – N | 120 | 143 |  |
|  | Mean (SD) | 3.0 (2.1) | 3.1 (2.0) | 0.05  (-0.29 to 0.40) |
|  | P-value | - | - | 0.765 |
|  | 6 months – N | 153 | 164 |  |
|  | Mean (SD) | 3.4 (2.1) | 3.2 (2.2) | -0.21  (-0.55 to 0.13) |
|  | P-value | - | - | 0.232 |
| Campaign to end loneliness scale | Baseline – N | 216 | 241 |  |
|  | Mean (SD) | 9.7 (3.1) | 9.8 (3.1) |  |
|  | 3 months – N | 136 | 154 |  |
|  | Mean (SD) | 8.7 (3.9) | 9.1 (3.8) | 0.34  (-0.51 to 1.19) |
|  | P-value | - | - | 0.435 |
|  | 6 months – N | 167 | 180 |  |
|  | Mean (SD) | 9.1 (3.8) | 9.0 (3.8) | -0.00  (-0.76 to 0.76) |
|  | P-value | - | - | 0.999 |
| Duke Social Support Scale | Baseline – N | 214 | 234 |  |
|  | Mean (SD) | 22.1 (4.4) | 22.2 (4.4) |  |
|  | 3 months – N | 117 | 131 |  |
|  | Mean (SD) | 21.8 (4.6) | 22.1 (4.6) | -0.21  (-0.97 to 0.55) |
|  | P-value | - | - | 0.582 |
|  | 6 months – N | 144 | 152 |  |
|  | Mean (SD) | 21.6 (4.6) | 22.4 (4.5) | 0.35  (-0.40 to 1.11) |
|  | P-value | - | - | 0.363 |
| Network responsiveness | Baseline – N | 213 | 233 |  |
| (CENS) | Mean (SD) | 3.47 (0.70) | 3.43 (0.75) |  |
|  | 3 months – N | 131 | 147 |  |
|  | Mean (SD) | 3.46 (0.83) | 3.35 (0.94) | 0.01  (-0.15 to 0.16) |
|  | P-value | - | - | 0.941 |
|  | 6 months – N | 165 | 177 |  |
|  | Mean (SD) | 3.33 (0.86) | 3.36 (0.89) | 0.02  (-0.13 to 0.16) |
|  | P-value | - | - | 0.833 |
| Access to collective | Baseline – N | 217 | 236 |  |
| efficacy (CENS) | Mean (SD) | 3.41 (0.80) | 3.30 (0.81) |  |
|  | 3 months – N | 136 | 150 |  |
|  | Mean (SD) | 3.35 (0.85) | 3.29 (0.91) | -0.03  (-0.21 to 0.15) |
|  | P-value | - | - | 0.761 |
|  | 6 months – N | 169 | 178 |  |
|  | Mean (SD) | 3.44 (0.87) | 3.32 (0.87) | -0.03  (-0.19 to 0.13) |
|  | P-value | - | - | 0.705 |
| Social support (SPS) | Baseline – N | 183 | 195 |  |
|  | Mean (SD) | 72.9 (6.8) | 73.3 (7.8) |  |
|  | 3 months – N | 110 | 129 |  |
|  | Mean (SD) | 75.5 (8.7) | 74.7 (8.8) | -0.45  (-2.35 to 1.45) |
|  | P-value | - | - | 0.644 |
|  | 6 months – N | 128 | 149 |  |
|  | Mean (SD) | 74.4 (8.6) | 74.5 (9.2) | 0.12 (-1.65 to 1.89) |
|  | P-value | - | - | 0.894 |

SD: standard deviation; CI: confidence interval.
