# Supplementary material for: Assessing a facilitated social network intervention for health outcomes in lonely and socially isolated people: the pragmatic, cluster-randomized PALS trial
Source: Front Public Health. 2026 Mar 30;14:1701579. doi: 10.3389/fpubh.2026.1701579 (PMC13073093; doi:10.3389/fpubh.2026.1701579)
Supplement: Supplementary file 3 [file Supplementary_file_3.docx]

**Supplementary File 3. Resource use analysis**

Tables reproduced from "[Social network intervention for loneliness and social isolation in a community setting: the PALS cluster-RCT](https://www.journalslibrary.nihr.ac.uk/phr/WTJH4379)" by Rebecca Band, Karina Kinsella, Jaimie Ellis, Elizabeth James, Sandy Ciccognani, Katie Breheny, Rebecca Kandiyali, Sean Ewings and Anne Rogers, licensed under [CC BY 4.0](https://creativecommons.org/licenses/by/4.0/deed.en).

**Table 1: Mean and sd of participant activities by group and timepoint**

|  | Usual Care | Intervention |
| --- | --- | --- |
| Mean number of activities at baseline | 2.28 (1.61) n=137 | 2.34 (1.55) n=149 |
| Mean number of activities at 3 months | 2.38 (1.41) n=88 | 2.57 (1.71) n=100 |
| Mean number of activities at 6 months | 1.97 (1.25) n=91 | 2.37 (1.66) n=107 |

Table reports all available cases

**Table 2: Descriptive statistics of costs by sector by group.**

|  | | Arm | | |
| --- | --- | --- | --- | --- |
|  |  | Control | Intervention | Total |
| Public sector costs^a^ (£) (3M and 6M) | Total | 84,404.70 | 106,429 | 190,834 |
|  | Mean | 691.84 | 744.26 | 720.13 |
|  | SD | 1,009.81 | 912.55 | 957.07 |
|  | N | 122 | 143 | 265 |
| Societal costs (complete case)^b^ (£) (3M and 6M) | Total | 32,289.40 | 44,396.50 | 76,686 |
|  | Mean | 566.48 | 652.89 | 613.49 |
|  | SD | 470.44 | 601.32 | 545.15 |
|  | N | 57 | 68 | 125 |
| ^a^ Includes NHS and PSS costs at 3 and 6 months (complete case) and intervention costs  ^b^ Includes NHS and PSS costs, informal care and activity data at 3 and 6 months (all complete case) | | | | |

**Table 3** **Mean and standard deviation of outcome measure by timepoint and group**

|  | SF6D | | ICECAP-A | |
| --- | --- | --- | --- | --- |
|  | Control | Intervention | Control | Intervention |
| Baseline | 0.648 (.141) | 0.650 (.155) | 0.714 (0.194) | 0.709 ((0.216) |
| Three months | 0.622 (.144) | 0.636 (.144) | 0.678 (0.219) | 0.712 (0.230) |
| Six months | 0.611 (.152) | 0.623 (.153) | 0.672 (0.216) | 0.686 (0.244) |
| QALYS | 0.315 (.069) | 0.319 (.071) | 0.348 (0.097) | 0.360 (0.109) |
