# Supplementary material for: Assessing a facilitated social network intervention for health outcomes in lonely and socially isolated people: the pragmatic, cluster-randomized PALS trial
Source: Front Public Health. 2026 Mar 30;14:1701579. doi: 10.3389/fpubh.2026.1701579 (PMC13073093; doi:10.3389/fpubh.2026.1701579)
Supplement: Supplementary file 4 [file Supplementary_file_4.docx]

Supplementary File 4: Net monetary benefit

**Figure 1: Primary analysis: net benefit by WTP.** Reproduced from "[Primary analysis: net benefit by WTP](https://www.journalslibrary.nihr.ac.uk/phr/WTJH4379)" by Rebecca Band, Karina Kinsella, Jaimie Ellis, Elizabeth James, Sandy Ciccognani, Katie Breheny, Rebecca Kandiyali, Sean Ewings and Anne Rogers, licensed under [CC BY 4.0](https://creativecommons.org/licenses/by/4.0/deed.en).


**Figure 2: Primary analysis: cost-effectiveness acceptability curve.** Reproduced from "[Primary analysis: CEAC](https://www.journalslibrary.nihr.ac.uk/phr/WTJH4379)" by Rebecca Band, Karina Kinsella, Jaimie Ellis, Elizabeth James, Sandy Ciccognani, Katie Breheny, Rebecca Kandiyali, Sean Ewings and Anne Rogers, licensed under [CC BY 4.0](https://creativecommons.org/licenses/by/4.0/deed.en).

**Figure 4: Secondary analysis: net benefit by WTP (imputed data).** Reproduced from "[Secondary analysis: net benefit by WTP (imputed data)](https://www.journalslibrary.nihr.ac.uk/phr/WTJH4379)" by Rebecca Band, Karina Kinsella, Jaimie Ellis, Elizabeth James, Sandy Ciccognani, Katie Breheny, Rebecca Kandiyali, Sean Ewings and Anne Rogers, licensed under [CC BY 4.0](https://creativecommons.org/licenses/by/4.0/deed.en).

**Figure 5:** **Secondary** analysis: CEAC (imputed data). **).** Reproduced from "[Secondary analysis: CEAC (imputed data).](https://www.journalslibrary.nihr.ac.uk/phr/WTJH4379)" by Rebecca Band, Karina Kinsella, Jaimie Ellis, Elizabeth James, Sandy Ciccognani, Katie Breheny, Rebecca Kandiyali, Sean Ewings and Anne Rogers, licensed under [CC BY 4.0](https://creativecommons.org/licenses/by/4.0/deed.en).
